# Implementation of an mHealth intervention to increase adherence to triage among HPV positive women with HPV—self-collection (ATICA study): post-implementation evaluation from the women's perspective

**DOI:** 10.1186/s12905-023-02475-0

**Published:** 2023-06-23

**Authors:** Melisa Paolino, Victoria Sánchez Antelo, Racquel E. Kohler, Kasisomayajula Viswanath, Silvina Arrossi

**Affiliations:** 1 Centre for the Study of State and Society, National Council for Scientific and Technical Research AR, Buenos Aires, Argentina; 2 Cancer Health Equity, Cancer Institute of New Jersey, Rutgers - the State University of New Jersey, New Jersey, USA; 3 Department of Social and Behavioral Sciences, Harvard T.H. Chan School of Public Health, Harvard University, Boston, USA

**Keywords:** Short text messages, HPV self-collection test, Cervical cancer prevention, Implementation science, Argentina

## Abstract

**Background:**

Low adherence to triage after positive screening is a widespread problem for cervical cancer screening programs in Low- and Middle-income Countries. Adherence to cytology-based triage can be challenging, especially among women with self-collected tests. SMS-based interventions are accepted by women and can increase screening uptake. The ATICA study was an effectiveness-implementation hybrid type I trial, combining a cluster randomized controlled trial (RCT) with a mixed-methods implementation evaluation involving quantitative and qualitative methods. Although the RCT provided evidence regarding the effectiveness of the SMS-based intervention, less is known about its acceptability, relevance, and usefulness from the women´s perspective.

**Methods:**

We carried out a cross-sectional study based on a structured questionnaire among HPV-positive women who were enrolled in ATICA's intervention group. We measured acceptability, appropriateness, and message content comprehension. Also, we evaluated if the SMS message was considered a cue to encourage women to pick up their HPV test results and promote the triage.

**Results:**

We interviewed 370 HPV-positive women. Acceptability of SMS messages among women who had received at least one message was high (97%). We found high levels of agreement in all appropriateness dimensions. More than 77% of women showed high comprehension of the content. Among women who received at least one SMS message, 76% went to the health center to pick up their results. Among those who got their results, 90% reported that the SMS message had influenced them to go. We found no significant differences in acceptability, appropriateness or message comprehension between women who adhered to triage and those who did not adhere after receiving the SMS messages.

**Conclusion:**

The intervention was highly acceptable, and women reported SMS was an appropriate channel to be informed about HPV test results availability. SMS was also a useful cue to go to the health center to pick up results. The implementation did not encounter barriers associated with the SMS message itself, suggesting the existence of other obstacles to triage adherence. Our results support the RCT findings that scaling up SMS is a highly acceptable intervention to promote cervical screening triage adherence.

## Background

The development of the human papillomavirus (HPV) test has changed the cervical cancer screening paradigm. It is a highly sensitive screening method [1–3] and it has been demonstrated to reduce cervical cancer incidence and mortality [2]. In addition, it allows women to self-collect samples, which increases screening coverage, especially among underscreened women [4, 5]. In an HPV-test-based program, triage tests are key to identify which HPV-positive women need triage, diagnosis and treatment procedures. Adhering to triage after a positive HPV test can be challenging, especially for women who performed self-collected tests because screening occurs outside the medical facility. After a positive screening result, patients must receive screening results, understand the meaning of positive results, and take action on that information to continue along the screening process to triage. Studies carried out in Argentina and other Latin American countries showed that women who had performed self-collection were less likely to complete follow-up procedures than women who performed clinician-collected tests [6–12]. mHealth interventions (i.e. the use of mobile technologies for public health) improve the link between users and health providers and have increased medication compliance, or adherence to medical visits via reminders for diverse health problems, and in different settings [13–21]. Regarding cervical cancer prevention, evidence has suggested that SMS-based interventions are highly accepted and they have been demonstrated to increase screening uptake [21–23]. To evaluate the effectiveness and implementation of a multi-component mHealth intervention to increase adherence to triage Pap among women with HPV-positive self-collected test, we conducted the ATICA Study (Application of Communication and Information Technologies to Self-Collection, for its initials in Spanish) [24, 25]. It was a hybrid type I trial, that combined a cluster randomized controlled trial (RCT) with a mixed-methods evaluation [24]. The multi-component mHealth intervention included: 1) one weekly SMS message (four-week period), notifying women that HPV results were available at the health center, 2) an SMS message sent to Community Health Workers (CHWs) to visit those women who, after 60 days from the HPV result, had not registered triage Pap [24]. The intervention was designed based on the Health Belief Model as described in other publications. We expected that the mHealth intervention acted as a specific cue to action and increase adherence to triage [26]. Final results of ATICA RCT showed that the multi-component mHealth intervention was effective to increase adherence to triage. In addition, weekly SMS messages also increased adherence to triage Pap at day 60 (54% of HPV-positive women in the intervention group versus 33% in the control group) [25].

Even though the SMS message was designed in formative research with women [26] and the RCT results showed that it was effective to increase adherence to triage compared to usual care [25], 46% of women of the intervention group did not have triage Pap in 60 days after receiving SMS messages [25]. Therefore, evaluating the intervention from the user´s perspective is important to identify potential implementation barriers that may have affected triage adherence (e.g. low acceptability, inappropriate fit, or confusing content). Indeed, an SMS message about a sexually transmitted infection such as HPV must be constructed and sent with caution. Patients’ privacy and confidentiality need to be protected and at the same time, the message must be short, easy to understand, and also culturally appropriate [26, 27]. Not considering the user’s perceptions about content, design, and context are documented barriers to the acceptability and effectiveness of SMS-based health interventions in other settings [20, 28, 29]. Although there is increasing evidence regarding the effectiveness of mHealth interventions for cancer prevention, less is known about acceptability, relevance, and usefulness from the user´s perspective.

In this paper we report results of a post-implementation evaluation in which we analyzed women’s perceptions about the first component of ATICA – the SMS message. We assessed acceptability, appropriateness, comprehension, and perception of the effect of the intervention on their adherence to triage. These implementation outcomes complement RCT results on effectiveness and implementation previously published [25, 30]. and will contribute to a deeper understanding of ATICA’s approach and identify the implementation factors that should be considered in future scale up.

## Methods

### Setting

This study took place in Jujuy province, which is in northwest Argentina. Majority (85%) of its population lives in urban areas and 69% are women. Out of the total province population, 45% had public health insurance [31]. In 2021, mobile phone penetration in urban areas was 90% [32].

The public health sector includes a network of public hospitals and primary health care centers which mostly provide care to informal economy workers and unemployed people who are not covered by social security or private insurance. For the uninsured, health services are provided free of cost. Around 700 CHWs visit approximately 110,000 households twice per year for health services such as height/weight measurements and child vaccination. HPV-testing has been the primary screening method for cervical cancer prevention since 2012 [7], targeting women aged 30 years and older attending the public health system. The screening protocol in Jujuy has been described elsewhere[7]. Briefly, HPV-positive women are triaged with cytology. Women with ASCUS + are referred to colposcopy and biopsy if needed. Women with histologically confirmed CIN2 + are referred for treatment. HPV-negative women are recommended re-screening in five years. In 2014, HPV self-collection offered by CHWs during home visits was introduced as a programmatic strategy to increase screening coverage. If a woman performs self-collection at home, she is instructed to go to the health center within 30 days to retrieve her results and, if her results are positive, then she must have triage cytology at the health center [33].

### Study design

The design and methods of the ATICA study have been extensively described elsewhere [24]. After the RCT endpoints were completed, a cross-sectional survey was conducted using a structured questionnaire among HPV-positive women who were enrolled in the intervention group.

### Data collection and sample

The list of HPV-positive women and their contact details were extracted from ATICA RCT database [24]. Of the 3241 women who participated in the intervention group, 445 had an HPV-positive result. All HPV-positive women from the intervention group were contacted by trained interviewers for an in-person/phone interview [24]. Interviews took place between December 2019 and October 2020. We interviewed 370 (83%) HPV-positive women; 59 women could not be reached, 3 had died and 13 refused to answer the survey.

The questionnaire included open-ended questions with dimensions related to women’s perceptions about the implementation of the SMS messages: acceptability, appropriateness, comprehension, and perception of the effect of the of the SMS message on their adherence to triage as described below.

### Outcome measures

#### Women’s characteristics

We extracted the following sociodemographic variables from the ATICA RCT database: Age; Area of residence (Urban/Rural); Education (Never went to school/primary incomplete, Primary complete/ secondary incomplete, and Completed secondary/tertiary incomplete/complete); Overcrowding (> 3 people/room); Household with children younger than 5 years-old (Yes/No); Health insurance (Public /Private or social security); Screening in the last 10 years (HPV test or Pap). We also measured variables related to the use of technology: shared phones with other family members (Yes/No); had a personal computer (Yes/No); had Internet access through their mobile phone (Yes/No); used social networks apps (e.g., Facebook, WhatsApp, etc.) (Yes/No).

#### Acceptability and appropriateness of the intervention

Following Proctor´s Taxonomy of Implementation Outcomes [34], we measured acceptability and appropriateness, which are essential to understand the success (or failure) of the implementation of an intervention from users’ perspective.

*Acceptability* was defined as the perception among women that the mHealth intervention (SMS message) is agreeable or satisfactory. We evaluated acceptability measuring the level of agreement with the statement: “An SMS message is a good communication channel to be informed that the HPV self-collection result is available at the health center”.

*Appropriateness* was defined as women´s perceived fit, relevance, or compatibility of the SMS message as a communication channel through which to be informed about the availability of the HPV self-collection result. As noted by Proctor et al., appropriateness is conceptually similar to acceptability, and the literature reflects overlapping terms when discussing these constructs [34]. Following Proctor’s Taxonomy, we preserved the distinction because a given intervention may be perceived as appropriate but not acceptable, and vice versa [35]. For example, the SMS message could be perceived as a good channel of communication but if its content is not appropriate for the target population, the intervention might fail. To evaluate appropriateness, we measured the level of agreement with statements related to different aspects of the intervention: usefulness, frequency and number of messages, confidentiality, personalization, credibility, and pertinence of content.

We used a 5-category response option to measure the level of agreement (Strongly agree, Agree, Neutral, Disagree, and Strongly disagree) with a set of statements related to acceptability and appropriateness dimensions (Table 1):

**Table 1:** Acceptability and appropriateness dimensions and statements

Dimension	Sub-dimension	Statements
Acceptability	N/A	**“**An SMS message is a good communication channel to be informed that the HPV self-collection result is available at the health center”
Appropriateness	Usefulness of the SMS as a reminder	“The SMS messages were useful to remind me to go get the result from the health center”
	Frequency and number of messages	“It bothered me that there were so many SMS messages”
		“The hours in which I received the SMS messages seemed adequate to me”
	Confidentiality concerns	I don't like to receive an SMS message informing me that the self-collection result is available as someone in my family can read it”
	Personalization of the SMS (Women’s Name)	“It bothered me that the SMS message had my name on it”
	Credibility (i.e., Trust in the SMS sender)	The number from which I received the SMS message made me suspicious”
	Pertinence of the content	“When I read the SMS message, I felt that it was intended for me”
		“The content of the SMS message seemed cold to me”
		“When I received the SMS message, I felt that the health center was taking care of me”
		“I was happy to have received the SMS message advising me that my result was already at the health center”

#### Comprehension

**Fig. 1:**
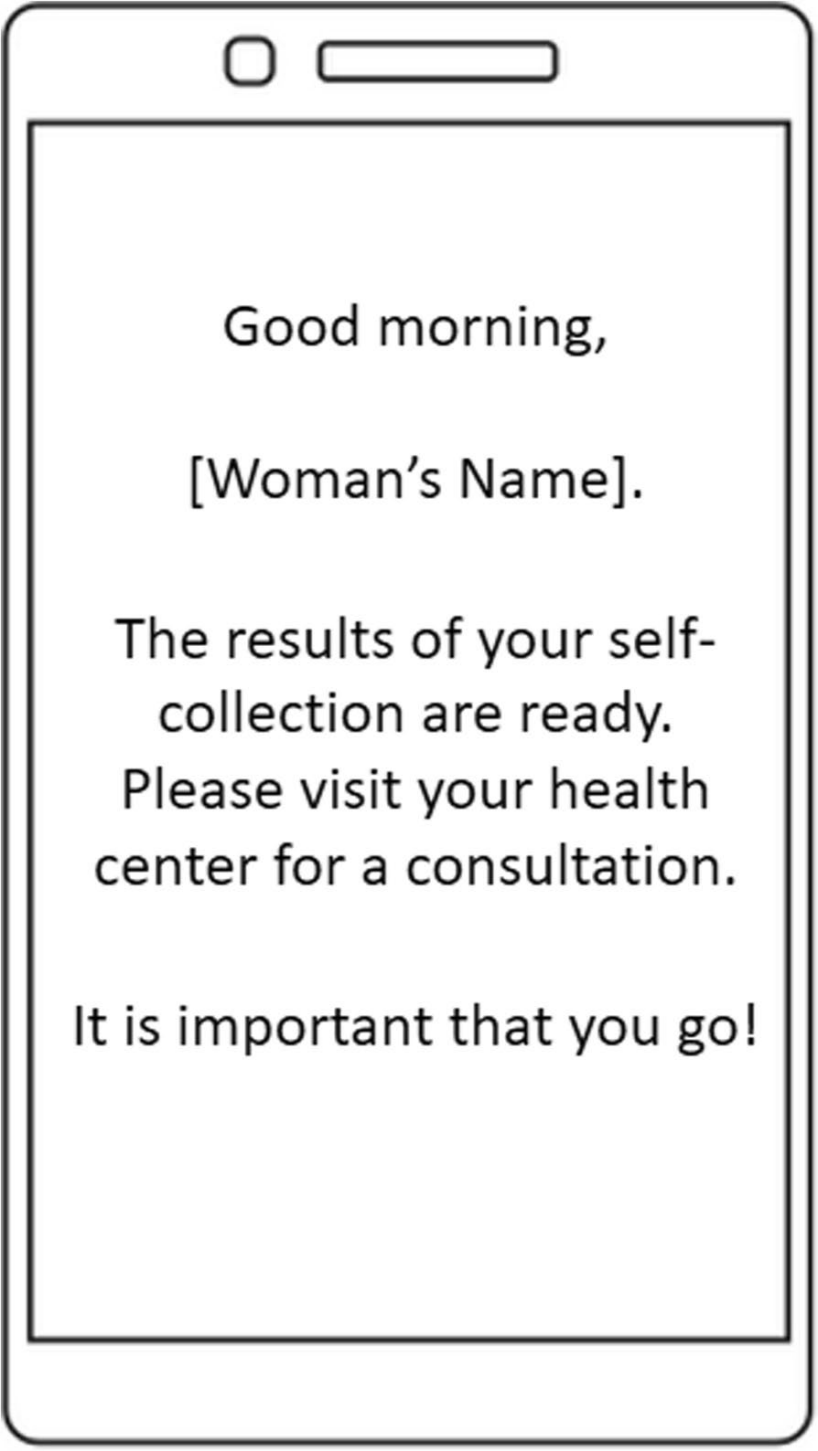
SMS message content

We evaluated comprehension of the SMS message content with multiple questions. First, we measured the level of agreement (Strongly agree, Agree, Neutral, Disagree, and Strongly disagree) with the statements: “The SMS content was confusing” and “The message was cut off”. Second, we asked women to describe the key ideas of the message: “What are the most important ideas in that text message?” (See SMS content in Fig. 1). Finally, we asked for suggestions to improve the content of the SMS message.

#### SMS message as a cue to action

Following the Health Belief Model, the SMS message was conceptualized as a specific cue to encourage women to pick up their HPV test results and promote the triage [26]. We evaluated the level in which the SMS message was considered cue to go to the health center and pick up the results with the following questions: picked up the results after receiving the SMS messages (yes/no), number of days between receiving the SMS message and going to the health center, and SMS message as a cue to help women to decide to go to the health center (yes/no).

The information about triage adherence at day 60 was extracted from RCT database.

### Data analysis

We conducted a descriptive analysis using frequencies and percentages for each variable. We also calculated the percentage of agreement (strongly agree/agree responses) with acceptability and appropriateness statements: number of answers strongly agree/agree out of the total of women who responded. For multiple-choice questions (e.g., variable "understanding of the message"), the percentage of each option was calculated as follows: number of times an option was chosen out of the total mentioned options. All percentages of agreement with acceptability, appropriateness and comprehension statements were calculated among women who remember received SMS messages.

We compared acceptability, appropriateness, comprehension, and perception of SMS messages as a cue to action by triage adherence. Association between variables was determined using Chi-squared tests. Significance was assumed at a two-sided value of p < 0.05. We used R statistical software (version 3.5.0) for all analysis.

## Results

### Women characteristics

Out of 445 HPV-positive women, 370 answered the survey (83%; *N* = 370/445). There were no significant differences regarding sociodemographic characteristics between women who responded to the survey and those who did not (Table 2).

**Table 2:** Characteristics of HPV-positive women enrolled in ATICA intervention by follow-up survey response

	**HPV-positive women surveyed**				***p*-value**
	**Responded**		**Did not respond**		
	**n**	**%**	**n**	**%**	
	**370**	**100.0**	**75**	**100.0**	
**Age**					
30–39	126	34.1	23	30.7	0.397
40–49	113	30.5	18	24.0	
50–64	72	19.5	20	26.7	
65 +	59	15.9	14	18.7	
**Area of residence**					
Urban	289	78.1	62	82.7	0.378
Rural	81	21.9	13	17.3	
**Education**					
Never went to school/ Primary (incomplete)	30	8.1	10	13.3	0.132
Completed Primary / Secondary (incomplete)	115	31.1	28	37.3	
Completed Secondary or higher	225	60.8	37	49.3	
**Overcrowding (> 3 people/room)**					
No	303	81.9	60	80.0	0.700
Yes	67	18.1	15	20.0	
**Household with children younger than 5**					
Yes	143	38.6	29	38.7	0.998
No	227	61.4	46	61.3	
**Health insurance**					
Public	310	83.8	61	81.3	0.603
Private/social security	60	16.2	14	18.7	
**Screening in the last 10 years (HPV test or Pap)**					
Yes	211	57.0	45	60.0	0.635
No	159	43.0	30	40.0	
**Shared phones with other family members**					
Yes	49	13.2	11	14.7	0.742
No	321	86.8	64	85.3	
**Mobile phone plan**					
Pre-paid plan	193	52.2	28	37.3	0.240
Monthly plan	177	47.8	46	61.3	
**Had Personal Computer (≥ 1 per household)**					
Yes	155	41.9	22	29.3	0.430
No	215	58.1	53	70.7	
**Mobile phone with internet access**					
Yes	326	88.1	61	81.3	0.112
No	44	11.9	14	18.7	
**Use of social networks**					
Yes	330	89.2	66	88.0	0.764
No	40	10.8	40	53.3	
**Triage Pap at 60 days**					
Yes	200	54.1	42	56.0	0.758
No	170	45.9	33	44.0	

Characteristics of interviewed women by triage adherence are shown in Table 3. Nearly two-thirds (64%) were aged 30–49, 78.1% lived in urban areas, and 60.8% had completed secondary or attended some higher education. Over half (57%) had been screened in the last 10 years. Most (88%) had a mobile phone with internet access and 89% used social networks. Few (13%) shared phone with family members.

**Table 3:** Characteristics of interviewed women

			**Pap at day 60**			
	**Total**		**Yes**		**No**	
	**n**	**%**	**n**	**%**	**n**	**%**
	**370**	**100**	**200**	**54.1**	**170**	**45.9**
**Age**						
30–39	126	34.1	72	36.0	54	31.8
40–49	113	30.5	52	26.0	61	35.9
50–64	72	19.5	40	20.0	32	18.8
65 +	59	15.9	36	18.0	23	13.5
**Area of residence**						
Urban	289	78.1	155	77.5	134	78.8
Rural	81	21.9	45	22.5	36	21.2
**Education**						
Never went to school / Primary (incomplete)	30	8.1	21	10.5	9	5.3
Completed Primary / Secondary (incomplete)	115	31.1	60	30.0	55	32.4
Completed Secondary or higher	225	60.8	119	59.5	106	62.4
**Overcrowding (> 3 people/room)**						
No	303	81.9	169	84.5	134	78.8
Yes	67	18.1	31	15.5	36	21.2
**Household with children younger than 5**						
Yes	143	38.6	75	37.5	68	40.0
No	227	61.4	125	62.5	102	60.0
**Health insurance**						
Public	310	83.8	172	86.0	138	81.2
Private/social security	60	16.2	28	14.0	32	18.8
**Screening in the last 10 years (HPV test or Pap)**						
Yes	211	57.0	126	63.0	85	50.0
No	159	43.0	74	37.0	85	50.0
**Shared phones with other family members**						
Yes	49	13.2	29	14.5	20	11.8
No	321	86.8	171	85.5	150	88.2
**Mobile phone plan**						
Pre-paid plan	193	52.2	110	55.0	83	48.8
Monthly plan	177	47.8	90	45.0	87	51.2
**Had Personal Computer (≥ 1 per household)**						
Yes	155	41.9	88	44.0	67	39.4
No	215	58.1	112	56.0	103	60.6
**Mobile phone with internet access**						
Yes	326	88.1	170	85.0	156	91.8
No	44	11.9	30	15.0	14	8.2
**Use of social networks**						
Yes	330	89.2	172	86.0	158	92.9
No	40	10.8	28	14.0	12	7.1
**Remembered receiving at least one SMS message**						
Yes	289	78.1	164	82.0	125	73.5
No	81	21.9	36	18.0	45	26.5

Seventy-eight percent (*N* = 289/370) of HPV-positive women remembered that they had received at least one SMS message from ATICA. This percentage was higher among women with Pap at day 60 (82% vs. 73%). Only 7% of women had not received the SMS messages due to problems with the registered phone number.

### Acceptability and appropriateness of the SMS message

Acceptability of SMS messages among women who remembered that they had received at least one SMS message from ATICA was high (97.2%, *n* = 278/286) with no significant differences between women with vs. without triage Pap at day 60 (Table 4).

**Table 4:** Level of agreement with acceptability, appropriateness, and comprehension statements

Dimension	Statements	Total		Adhered to Triage Pap at 60 days	
				**Yes**	**No**
		**N **^**a**^	**% Agree**	**% Agree**	**% Agree**
Acceptability	**“**An SMS message is a good communication channel to be informed that the HPV self-collection result is available at the health center”	286	97.2	97.5	97.6
Appropriateness	“The SMS messages were useful to remind me to go get the result from the health center”	285	98.9	98.4	99.4
	“It bothered me that there were so many SMS messages””	279	7.2	5.0	9.8
	“The hours in which I received the SMS messages seemed adequate to me”	273	96.3	95.3	93.3
	“When I read the SMS message, I felt that it was intended for me” *	**281**	**92.2**	**94.4**	**87.8**
	“The content of the SMS message seemed cold to me”	280	19.3	17.0	22.0
	“When I received the SMS message, I felt that the health center was taking care of me”	285	96.1	96.9	95.2
	“I was happy to have received the SMS message advising me that my result was already at the health center”	279	96.1	95.0	93.5
	“It bothered me that the SMS message had my name on it”	284	4.2	4.3	4.0
	"I don't like to receive a SMS message informing that the self-collection result is available as someone in my family can read it”	284	10.6	7.5	14.5
	The number from which I received the SMS message made me suspicious” *	**280**	**23.6**	**19.9**	**31.7**
Comprehension	"The message was cut off”	283	4.6	4.4	4.8
	The SMS message was confusing	283	17.3	15.6	19.5

^a^ Total women who respond this question

^*^p-value < 0.05

All positive dimensions related to appropriateness of the SMS message also showed a high level of agreement and the negative dimensions had low agreement (Table 4).

Nearly all (98.9%, *N* = 282/285) of women agreed that the SMS messages were useful to remind them to go to the health center. In addition, most women did not report problems with the number of SMS messages they received and found the message timing to be appropriate.

The relevance of the SMS content was also high, as more than 90% of interviewed women agreed with positive statements about the SMS content. Ninety-two percent of women felt that the SMS was intended for them. Importantly, fewer women who did not attend triage agreed with this statement (87.8% vs. 94.4%, p = 0.049). In addition, only 19.3% agreed with the statement “the content of the SMS messages seemed cold to me”. The personalization of the SMS message was positively valued: only 4.2% were bothered by having their name in the SMS.

Nearly a quarter (23.6%) of the respondents questioned the number from which they received the message, which was higher among women who did not adhere to triage Pap than those who did (31.7% vs. 19.9%, p = 0.003).

### SMS message comprehension and preferences

The comprehension of the SMS message content was high with more than 77% of open-ended mentions indicating that women understood the SMS key ideas (*That the result is available. I have to go to the health center. That it is important that I go. I have to go)*. Seventeen percent of women reported that the content was confusing. There were no significant differences between women with vs. without triage Pap (Table 4).

88.1% of women who received the SMS message would not change its content.

### SMS as a cue to action

Among the women who remembered receiving at least one SMS message, 76% (220/289) went to the health center to pick up their result. Almost a quarter of women did not go to the health center. The main reasons for this were: CHWs or a Health professional informed the results during a home visit; working reason; childcare and domestic workload; a health condition that needed attention; they thought that a CHW was going to call or visit them, and they did know where to pick it up.

Among those who picked up results, 90% (*n* = 199/220) reported that the SMS message had influenced them to go. Although this percentage was higher (91.4% vs. 88.4%) among women who adhered to triage Pap, it was not significantly different (p = 0.485). Sixty-nine percent of women went to the health center within 7 days after receiving the message, including 7.5% who went on the same day they received the SMS. The median time to go to the health center was 3.5 days after receiving the SMS.

Among the women who went to the health center to pick up HPV test results, 20.9% (*n* = 46/220) did not know the HPV result in the first attempt. The main reasons for this were: “the hospital had not sent the results to the health center” (39%), “the person who delivers the results (e.g. gynecologist) was not present in that moment (23%), and “additional appointment needed” (12.2%). Among these women, 76% (*n* = 35/46) returned at least once (average 2 times; range 1–7 times).

## Discussion

To our knowledge this is the first study that evaluated implementation outcomes of a mHealth intervention which effectively increased adherence to triage Pap among HPV-positive women in a Latin American country. Our post-intervention study showed high acceptability among women. All dimensions related to appropriateness also reported a high level of agreement: most women mentioned that the intervention was useful, positively valued the content, and found the number of messages and their timing were adequate. In addition, the comprehension of the SMS message content was high, and most women perceived the SMS message as a tool that influenced them to go to the health center to get their results. Findings showed high acceptability, appropriateness, and comprehension of the SMS message among HPV-positive women with no significant differences by triage adherence after receiving the SMS messages. These results are important as lack of comprehension of the SMS, as well as its low acceptability by the target population have been indicated in the literature as a main obstacle for effectiveness of mHealth strategies. These findings provide a comprehensive understanding of the effectiveness reported in ATICA trial [25], and could contribute to future scale up of the intervention.

High acceptability of an mHealth intervention is key to understanding its success [18]. Women’s acceptability of the intervention was high. Most considered that an SMS message was a good communication channel to be informed that the HPV self-collection result was available at the health center. The high acceptability reported in our study is consistent with results from other studies that showed that mobile interventions were an acceptable way of providing reminders for medication or appointments [35]. This high acceptability among women in ATICA is related to the women´s positive perceptions about the appropriateness of the message. Regarding the pertinence of content, most women mentioned that the health center was taking care of them and felt that the message was intended for them. However, the percentage of women who felt that the message was intended for them was slightly lower among women without triage. Other studies showed that participants indicated that the tone and content of the message could influence their acceptance and their engagement with the messages [36–40]. The SMS content and tone were carefully designed and pilot tested [26], which are key elements for the success of mHealth interventions [35, 41]. In addition, personalization of the message, including women´s names, was accepted by most women. ATICA results are similar to other studies that evaluated SMS-based health promotion intervention in which personalized SMS messages (e.g. with the recipient’s name) have been associated with greater intervention efficacy [35, 41]. High acceptability and appropriateness of the intervention are consistent with the effectiveness proved in the RCT. Our results on effectiveness and implementation showed that SMS messages could be a good option to send health information related to the availability of HPV results in low and middle resource settings.

Legitimacy of the sender is key to engagement with SMS-based interventions [36]. In our evaluation, we observed that mistrustful of the sender was higher among women without triage (32% vs. 20%). Results of ATICA´s formative research highlighted the need for messages to come from a trusted source. Similar recommendations have been made in a study on HIV testing among African immigrants in the United Kingdom [42]. Several studies have found that participants’ perceptions of the message source or sender could influence their perception of the intervention’s credibility and value [42–45]. Many studies suggest that the sender should be known and identifiable, otherwise recipients were more likely to ignore or delete the message [40, 43]. These results underscore that a trustworthy sender is a key element to be considered in future scale up to engage the recipient and to achieve high adherence to triage.

Low comprehension of the content of the message could be a potential barrier to the effectiveness of an SMS-based intervention [46]. In our study, most women mentioned that the message was clear and correctly interpreted its meaning. Our results support others’ findings on the importance of a careful development of message content [40, 47, 48] with attention to use of simple and clear language [49–51]. A study conducted in Chile about SMS preferences of under-screened women showed that clarity and simplicity of the message were very important for them [27]. A systematic review of qualitative studies about user´s perceptions of and experiences with mHealth interventions, showed that participants wanted short and concise messages that were easy to understand [35]. Thus, our results emphasized the need to adapt SMS content to the specific cultural context and intervention goals in order to guarantee high acceptability, effectiveness, and scalability of SMS-based health interventions.

Privacy and confidentiality issues, such as disclosing a result to a third-person in shared cellphones, are paramount for SMS-based health intervention [52, 53]. Several authors have noted that confidentiality is a challenge for SMS delivered to shared mobile phones to promote the treatment of sexually transmitted infections. In studies on sexually transmitted infections and SMS interventions, the main concern mentioned regarding confidentiality was the danger of disclosing results among relatives [54, 55]. In our study, only 10% of women did not like the SMS because a family member might read it, though there were not differences between women with and without Pap at day 60. These results suggest SMS message privacy was not raised as a serious concern. Although our protocol initially stipulated that the SMS message would include the HPV result, our previous formative research indicated that women preferred not to receive it, as they raised concerns about confidentiality, and considered that result delivery was a responsibility of a health professional. Using neutral language and tailoring the content are strategies that could contribute to dealing with confidentiality and privacy issues [35]. In our study, the SMS message only mentioned the term “self-collection”, and informed women about the test result availability and the importance of attending the health centre [24–26]. Omitting the result and the term HPV-testing in the SMS message might have reduced worries related to privacy and increased the intervention acceptability.

Several studies have shown the effectiveness of SMS-based strategies as reminders to increase adherence to medical visits [35]. In our study almost 100% of women considered that the SMS was useful to remind them to go to the health center to pick up results and 90% of women who went to the health center to pick up the result mentioned that the SMS message had influenced them to go. In addition, 50% of women went to the health center promptly—less than 4 days after receiving the message—and in some cases, the same day they received the SMS message. These results complement the ones related to the effectiveness reported in ATICA trial. The intervention significantly decreased the time to triage, showing that the triage level of 50% was reached around day 50 in the intervention group and around day 100 in the control group [25]. SMS works as a cue to action and improves adherence to triage, not only increasing the number of women that access to it but also reducing access time. This reduction could contribute to improving time to access to other steps of cancer care continuum such as diagnosis and treatment.

Our results showed high acceptability, appropriateness, and comprehension of the SMS message with no significant differences by triage Pap adherence at day 60 after receiving the SMS messages. These results indicate that the implementation of the intervention did not encounter barriers associated with the characteristics of the SMS message itself, suggesting the existence of other barriers to triage adherence. In fact, around 20% of women who went to the health center after receiving the SMS did not know the HPV result in the first attempt due to institutional barriers. Several studies showed that main barriers to follow up among women with positive screening results were related to institutional barriers, such as delays in result delivery or not receiving results, lack of appointments, or lack of human resources to take the samples [56, 57]. The use of a highly acceptable, low cost mHealth strategy, like ATICA’s SMS messages, could facilitate communication between women and providers and improve this critical step (i.e., delivery of results) in the cancer care continuum. However, to improve its effectiveness, the SMS-based intervention must be accompanied by improvements at the institutional level to guarantee timely access to triage, colposcopy, and treatment if needed.

Like all studies, there are some limitations to this analysis that must be taken into account. Due to COVID-19 pandemic the field work was interrupted, and it was not possible to contact all HPV positive women. However, sociodemographic characteristics of responding and non-responding women were similar. In addition, a low proportion of women were interviewed by phone between September and October 2020 with potential recall bias. It is also possible that courtesy bias could have resulted in an over-estimation of the acceptability of the SMS strategy.

## Conclusion

Results from this post-intervention survey showed that the ATICA mHealth intervention was highly accepted among the target population. The intervention was perceived as an appropriate communication channel to be informed about HPV test result availability and as a useful cue to go to the health center to pick up results. Our findings contribute to a more comprehensive understanding of the effectiveness achieved in the ATICA RCT and provided evidence that could be useful for scaling up the intervention in similar settings.

## Data Availability

De-identified individual participant data on which summary statistics and tables are based will be made available from the point of, and up to five years after the acceptance for publication of the main findings. These data can be requested to the Principal Investigator (Dr. Silvina Arrossi) and only under a data-sharing agreement. Other materials such as forms and questionnaires will be made freely available upon request which can be made to the Principal Investigator.

## Abbreviations

ASCUS +: Atypical Squamous Cells of Undetermined Significance or worse.
CHWs: Community Health Workers
CIN2 +: Cervical Intraepithelial Neoplasia grade 2 or worse.
HPV: Human papillomavirus
LMIC: Low- and Middle-Income Countries
RCT: Cluster randomized controlled trial
SMS: Short messaging service
